# Postoperative outcomes of inguinal hernia repair performed by surgical residents in routine clinical practice: a retrospective single-center analysis

**DOI:** 10.1515/iss-2025-0028

**Published:** 2025-09-18

**Authors:** Helga Oehler, Kim L. Zerr, Hanan El Youzouri, Ursula Pession, Wolf O. Bechstein, Armin Wiegering, Teresa Schreckenbach

**Affiliations:** Department of General, Visceral, Transplantation, and Thoracic Surgery, 9173 Frankfurt University Hospital and Clinics, Goethe University Frankfurt, Frankfurt/Main, Germany

**Keywords:** inguinal hernia repair, surgical residents, teaching operation, patients’ safety

## Abstract

**Objectives:**

Inguinal hernia repair is one of the most common surgical procedures performed worldwide. As part of training, surgical residents perform these procedures, which raises questions about the safety and efficacy of their involvement compared to specialists. This study compares the postoperative outcome and complications of inguinal hernia repairs conducted by residents under supervision with those performed by board certified surgeons independent of the method of surgery.

**Methods:**

This retrospective cohort study was performed at a single tertiary care center. 388 patients undergoing inguinal hernia repair, aged 18 years and older were included. Data were collected from 2014 to 2020.

**Results:**

The techniques used for the hernia repair were open repair using the Lichtenstein technique (n=159) and the transabdominal preperitoneal (TAPP) mesh technique (n=228). The hernia repairs were mostly performed as an open procedure by residents (n=72; 45 %), whereas board-certified surgeons mostly operated laparoscopically (n=190; 83 %). Surgical trainees had significantly longer operation times when performing a laparoscopic procedure than attending surgeons (means: 94 vs.70 min; *p*<0.001). However, there was no difference in operation time when an open procedure was performed (*p*=0.988). Univariate analyses revealed no influence of the level of training on overall postoperative morbidity (*p*=0.138) but showed that the TAPP technique was a protective factor for postoperative complications (*p*<0.001).

**Conclusions:**

Resident participation in inguinal hernia repair is safe and does not increase postoperative complications. In the tertiary care center, residents primarily performed open repairs, while board-certified surgeons favored the minimally invasive approach. Both techniques proved to be safe under appropriate supervision. These findings highlight the feasibility of structured surgical training in routine clinical practice, ensuring patient safety while maintaining high educational standards.

## Introduction

Inguinal hernia repair is one of the most common surgical procedures performed worldwide and the lifetime risk for men developing the condition is around 25 % [1]. Around 250,000 inguinal hernia repairs are performed in Germany each year [2]. Different techniques are used, and the choice depends on the age of the patients, symptoms, size of the hernia, and preference of the surgeon [3], 4]. Two of the most common techniques are the open Lichtenstein repair and the laparoscopic transabdominal preperitoneal (TAPP) patch technique [5], 6].

Due to the frequency of the condition and the operation, it is important for surgical trainees to learn about the diagnosis and treatment of inguinal hernias early on in their training. Inguinal herniotomy using the Lichtenstein technique is often considered as an initial procedure for hernia surgery and is also included in the list of procedures required for surgical board certification in Germany. However, it involves considerable risks, such as damage to the spermatic cord or testicular blood supply and the development of chronic pain [7], 8]. In addition, minimally invasive treatment of the inguinal hernia is increasingly preferred whenever possible, especially among younger adults [9], 10]. This is seen as more demanding than open treatment, so it is usually taught later during surgical training [11].

Hospitals specializing in hernia surgery should ideally provide both minimally invasive and open approaches for inguinal hernia repair to ensure comprehensive patient care and individualized treatment selection [12]. The availability of both techniques not only allows surgeons to tailor the procedure to patient-specific factors but also has direct implications for surgical education. To become well rounded and competent in hernia surgery, residents must be adequately trained in both techniques and gain experience in the nuances, benefits, and limitations of each approach [13]. This ensures that future surgeons will be well prepared to manage a wide range of clinical scenarios and can offer patients the most appropriate surgical option.

However, in times of increasing demands for patient safety, quality management, and cost efficiency, the question of whether the involvement of junior doctors in operations is safe and efficient is increasingly coming into focus. Studies have already shown that the involvement of junior doctors in operations often leads to longer operating times [11], 14]. Extended operating times can result in an increased rate of surgical-site infections [15]. Particularly in connection with mesh implants, this could result in an increase in mesh infections and thus postoperative morbidity, as shown for other implants [16]. Therefore, in most countries, a specialist standard applies, which means that all operations must be performed to the standard of a board-certified surgeon. This is usually ensured by surgical trainees operating under supervision and not alone.

The aim of this retrospective study was to evaluate the postoperative outcomes of inguinal hernia repair performed by surgical residents in comparison to board-certified surgeons. Focus was given to postoperative morbidity in a real-world clinical setting. To ensure a representative depiction of routine surgical practice, the analysis included both hernia repair techniques performed (the Lichtenstein procedure and TAPP), as well as recurrent and bilateral hernias without differentiating between surgical approaches.

## Materials and methods

### Patient demographics and clinical data

This retrospective cohort study was performed at the University Hospital Frankfurt and included 388 patients undergoing inguinal hernia repair (open and laparoscopic). The data were collected from 2014 to 2020. Data were analyzed in accordance with the STROBE guidelines [17]. The included patients were undergoing hernia repair for primary or recurrent and one-sided or bilateral inguinal hernia. Patients with a femoral hernia were excluded. The data recorded included clinical patient data such as age, sex, body mass index (BMI in kg/m^2^), comorbidities (coronary artery disease, chronic obstructive pulmonary disease, liver cirrhosis, renal failure, diabetes mellitus and dementia), American Society of Anesthesiologists (ASA) score, risk factors (such as smoking, viral hepatitis, HIV, and alcoholism), and prostatic cancer in anamnesis.

The study was approved by the Ethics Committee of the Doctoral Committee of the Department of Medicine at the Goethe University Frankfurt (No. 2022/788).

### Surgeons and surgical procedures

All residents and board-certified surgeons working in the department were included. We defined two groups of surgeons: residents (group A) and board-certified surgeons (group B). Surgical trainees ranged from postgraduate year (PGY) 1 to PGY 6. The number of procedures performed per trainee ranged from 1 to 20. The mean number of procedures performed per surgical trainee was 6.9. The number of procedures performed by each trainee was closely related to the length of time they had been employed at the surgical department.

Patients with private health insurance were generally operated on by consultant surgeons. The hernia-repair techniques included in this study were either an open Lichtenstein procedure or minimally invasive TAPP. The choice between Lichtenstein and TAPP procedure was based on a multifactorial clinical assessment. Key determinants included patient-specific factors such as the presence of comorbidities (e.g., liver cirrhosis), prior abdominal or pelvic surgeries, recurrent hernias, and overall surgical fitness. The Lichtenstein technique was also preferred in patients who refused general anesthesia. Notably, the selection of the Lichtenstein approach was not driven by educational or training considerations, but rather by individual clinical indications.

Data about duration of surgery, intraoperative complications, and hospital stay were collected.

### Complications

Complications were divided into intraoperative complications and postoperative complications. Intraoperative complications included bowel injury and injury of the spermatic cord or testicular vessels (in male patients). Postoperative complications included surgical site infections (SSIs), hematoma, seroma, numbness, chronic pain, or reoperation. Complications were graded according to the Clavien–Dindo classification (CDC) [18].

### Study endpoints and statistical analyses

The primary outcome was morbidity according to the CDC with special attention paid to the surgeon’s level of training. The secondary endpoints were operating time, length of hospital stay, and recurrence rate. All statistical analyses were performed using IBM SPSS Statistics for Windows (version 29.0; IBM, Chicago, IL, USA). Categorical variables were described as frequencies and percentages. Continuous variables were presented as the mean and standard deviation (SD). Categorical variables were compared using the chi-squared (χ^2^)-test or Fisher’s exact test as appropriate. Cramer’s *V* was used as a measure of the strength of association between categorical variables. Continuous variables were compared using a one-way analysis of variance (ANOVA).

Logistic regression analysis was used to identify factors associated with postoperative complications with CDC grades of ≥3. The following parameters were included in the univariable logistic regression analysis: age, body mass index (BMI), smoking status, ASA score, cardiovascular disease, diabetes mellitus, chronic obstructive pulmonary disease (COPD), prostatectomy, duration of surgery, bilateral hernia, and level of training. The results were expressed as odds ratios (ORs) with 95 % confidence intervals (CIs). A p-value of <0.05 was considered statistically significant in all tests. A multivariate analysis was not carried out, as only one factor was statistically significant in the univariate analysis.

## Results

### Patient characteristics

**Figure 1:**
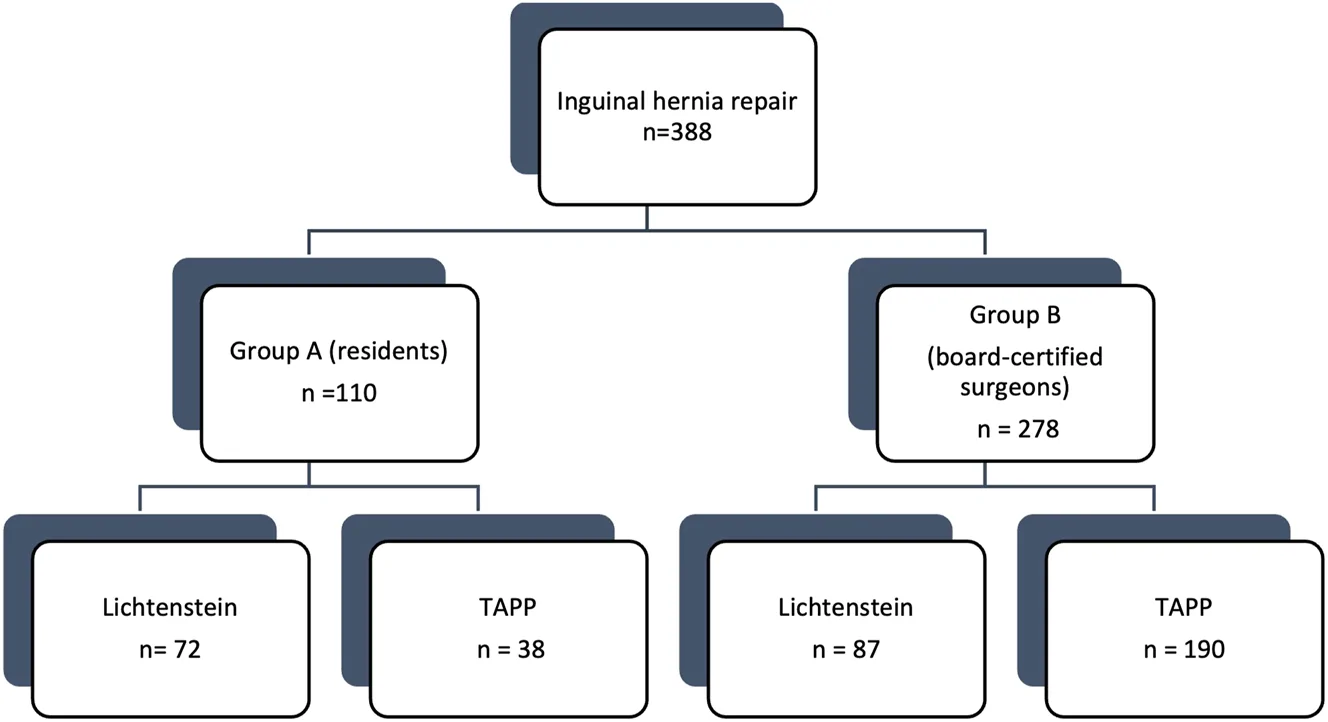
Study flow chart.

We recorded 388 hernia-repair surgeries between 2014 and 2020 in the department. Of those surgeries, 228 were laparoscopic (TAPP), and 159 were open (Lichtenstein) (Figure 1). The mean age of the study population was 58 ± 17 years, 367 patients were male (94.6 %), and the mean BMI was 25 ± 4 kg/m^2^. Furthermore, 155 patients (39.9 %) had risk factors, including smoking (25.3 %), viral hepatitis (3.4 %), HIV (2.3 %), and alcohol consumption (9 %). 268 patients had an ASA score of ≤2 (69.8 %).

There were statistically significant more female patients in group B (p=0.035). Furthermore, COPD was significantly more common in group A (p=0.009). Apart from those parameters, the groups did not show statistically significant differences in age (p=0.613), BMI (p=0.468), cardiovascular disease (p=0.462), liver cirrhosis (p=0.173), diabetes mellitus (p=0.324), and ASA score (p=0.315). The details are shown in Table 1.

**Table 1:** Patient characteristics.

	Total (%) n*=*388	Group A (%) n*=*110	Group B (%) n*=*278	Pearson chi-squared, *df*	Cramer’s *V*	p-Value
Gender				3.874 (1)	0.100	0.035^a^
Male	367 (94.6)	108 (98.2)	259 (93.2)			
Female	21 (5.4)	2 (1.8)	19 (6.8)			
Comorbidities						
Cardiovascular disease	179 (46.1)	54 (49.1)	125 (45.0)	0.540 (1)	0.037	0.462
COPD	18 (4.6)	10 (9.1)	8 (2.9)	6.877 (1)	0.133	0.009
Liver cirrhosis	19 (4.9)	8 (7.3)	11 (4.0)	1.861 (1)	0.069	0.173
Renal failure on dialysis	4 (1.0)	1 (0.9)	3 (1.1)	0.022 (1)	0.008	0.681^a^
Without dialysis	28 (7.2)	10 (9.1)	18 (6.5)	0.806 (1)	0.046	0.369
Diabetes mellitus	40 (10.3)	14 (12.7)	26 (9.4)	0.971 (1)	0.050	0.324
Dementia	6 (1.5)	3 (2.7)	3 (1.1)	1.406 (1)	0.060	0.224^a^
Prostatic cancer in the last 5 Years						
Yes	17 (4.4)	4 (3.6)	13 (4.7)	0.203 (1)	0.023	0.445^a^
Prostatectomy						
Yes	15 (3.9)	4 (3.6)	11 (4.0)	0.022 (1)	0.007	0.573^a^
Risk factors						
Smoking	98 (25.3)	26 (23.6)	72 (25.9)	0.214 (1)	0.023	0.644
Viral hepatitis	13 (3.4)	4 (3.6)	9 (3.2)	0.039 (1)	0.010	0.529^a^
HIV	9 (2.3)	3 (2.7)	6 (2.2)	0.113 (1)	0.017	0.493^a^
Alcoholism	35 (9.0)	11 (10.0)	24 (8.6)	0.179 (1)	0.022	0.672
ASA score (n=384)				1.008 (1)	0.051	0.315
ASA 1–2	268 (69.8)	72 (66.1)	196 (71.3)			
ASA≥3	116 (30.2)	37 (33.9)	79 (28.7)			

	**Total n=388**	**Group an n=110**	**Group B n=278**	***F***	**p-Value**	

Age in years (mean ± SD)	58 ± 17	59 ± 18	58 ± 16	0.256	0.613	
BMI (kg/m^2^) (mean ± SD)	25 ± 4	25 ± 5	25 ± 4	0.528	0.468	

COPD, chronic obstructive pulmonary disease; BMI, body mass index; SD, standard deviation; HIV, human immunodeficiency virus; ASA, American Society of Anesthesiologists. The chi-squared test was used to analyze the categorial variables. Pairwise comparisons between the groups were made with Bonferroni corrections. For continuous variables, one-way ANOVA analysis with Bonferroni correction was performed. ^a^Fisher’s exact test was used.

### Surgical details

Total of 354 (91.2 %) patients had a unilateral hernia, and 32 patients (14.0 %) had a bilateral hernia. Of these latter, 26 patients were operated on by board-certified surgeons (group B). In 5 cases (10.9 %), a recurrent hernia was involved. 228 patients (58.8 %) underwent TAPP, and 159 patients (41.0 %) underwent Lichtenstein repair. Lichtenstein repair was performed significantly more often by residents (p<0.001), and TAPP surgeries were performed significantly more often by board-certified surgeons (p<0.001).

There was a significant difference between the groups in operation time when a unilateral TAPP was performed (group A, 94 ± 31 min; group B, 70 ± 23 min; p<0.001). However, there was no difference in the duration of surgery when a Lichtenstein repair was performed (p=0.988). Furthermore, there was no difference in the length of hospital stay between the groups (p=0.793).

Intraoperative complications occurred in 4 cases (1.0 %), all within the consultant-led group; however, the difference in complication rates between groups was not statistically significant. Postoperative complications occurred in 38 cases (9.8 %), and there was no overall difference between the groups in this regard as well (p=0.058). The most common postoperative complications in the trainee group were hematomas (5.5 %) and surgical site infections (3.6 %). In the consultant-led group, hematomas were also the most frequent postoperative complication, occurring in 3.6 % of cases. More details are shown in Table 2. Detailed analysis of complications also showed no significant differences. Minor complications (CDC 1 and 2) occurred in 31 patients (8.0 %), and major complications (CDC≥3) occurred in seven patients (1.8 %).

**Table 2:** Surgical details.

	Total (%) n=388	Group A (%) n*=*110	Group B (%) n*=*278	Pearson chi-squared, *df*	Cramer’s *V*	p-Value
Recurrent hernia					0.116	0.373^a^
Yes	5 (1.29)	2 (1.82)	3 (1)			
Surgical technique Lichtenstein	159 (41.0)	72 (65.5)	87 (31.3)	38.024 (1)	0.313	<0.001
TAPP	228 (58.8)	38 (34.5)	190 (68.3)	37.157 (1)	0.309	<0.001
TAPP side				0.116 (1)	0.023	0.733
Unilateral	196 (86.0)	32 (84.2)	164 (86.3)			
Bilateral	32 (14.0)	6 (15.8)	26 (13.7)			
Drainage				0.770 (1)	0.045	0.380
Yes	25 (6.4)	9 (8.2)	16 (5.8)			
Patients with intraoperative complications				1.599 (1)	0.064	0.262^a^
Yes	4 (1.0)	0 (0)	4 (1.4)			
Intraoperative complications bowel injury	2 (0.5)	0 (0)	2 (0.7)	0.795 (1)	0.045	0.513^a^
Injury of spermatic cord	2 (0.5)	0 (0)	2 (0.7)	0.795 (1)	0.045	0.513^a^
Patients with postoperative complications				3.924 (1)	0.101	0.058
Yes	38 (9.8)	16 (14.5)	22 (7.9)			
Postoperative complications						
Surgical site infection	8 (2.1)	4 (3.6)	4 (1.4)	1.885 (1)	0.070	0.230^a^
Hematoma	16 (4.1)	6 (5.5)	10 (3.6)	0.688 (1)	0.042	0.407
Seroma	5 (1.3)	2 (1.8)	3 (1.1)	0.338 (1)	0.030	0.625^a^
Others	12 (3.1)	5 (4.5)	7 (2.5)	0.677 (1)	0.042	0.531^a^
Reoperation				1.599 (1)	0.064	0.581^a^
Yes	4 (1.0)	0	4 (1.4)			
Clavien–Dindo classification				7.116 (4)	0.135	0.132
CDC I	19 (4.9)	8 (7.3)	11 (4.0)			
CDC II	12 (3.1)	7 (6.4)	5 (1.8)			
CDC III	6 (1.5)	1 (0.9)	5 (1.8)			
CDC IV	1 (0.3)	0	1 (0.4)			
CDC V	0	0	0			
Other complications						
Persistent pain>3 months	5 (1.3)	1 (0.9)	4 (1.4)	0.174 (1)	0.021	0.562^a^
Numbness>3 months	1 (0.3)	1 (0.9)	0	2.534 (1)	0.081	0.284
Recurrent hernia after last operation	8 (2.1)	1 (0.9)	7 (2.5)	1.010 (1)	0.051	0.450^a^

	**Total n*=*388**	**Group A** **n*=*110**		**Group B** **n*=*278**	**F-value**	**p-Value**

TAPP operation time in minutes unilateral (mean ± SD)	74 ± 26	94 ± 31		70 ± 23	26.279	**<0.001**
TAPP operation time in minutes bilateral (mean ± SD)	113 ± 40	126 ± 26		110 ± 43	0.741	0.396
Lichtenstein operation time in minutes unilateral (mean ± SD)	67 ± 31	67 ± 24		67 ± 36	0.000	0.988
Hospital stay in days (mean ± SD)	2 ± 2	2 ± 1		2 ± 2	0.069	0.793

The chi-squared test was used to analyze the categorical variables. If appropriate, Fisher’s exact test was used (marked with^a^). Pairwise comparison between groups was done with Bonferroni correction. For continuous variables, one-way ANOVA with Bonferroni correction was performed.

No patient died from complications of inguinal hernia repair. As some patients developed more than one complication, the differentiated analysis of the complications showed 14 cases of hematoma (4.1 %), 8 cases (2.1 %) of SSIs, and 5 cases of seroma (1.3 %). Other complications included early recurrences due to mesh dislocation (n=2), epididymitis (n=1), funiculitis (n=1), urinary infections (n=4), paralytic ileus (n=2), obstructive ileus (n=1), and peritonitis following bowel perforation (n=1). Chronic pain after 3 month was documented in five patients (1.3 %). The hernia recurred in eight patients (2.1 %) during the follow-up period. No statistical differences were observed between the groups.

### Logistic regression analysis

Based on the univariable logistic regression analysis, only TAPP was found to be a protective factor for any postoperative complication (OR 0.247; p<0.001). Other risk factors were not identified. The level of surgical training did not influence the postoperative complication rate. Details of the analysis are shown in Table 3.

**Table 3:** Univariable logistic regression analysis for risk factors for any complications according to CDC.

	Any complication according to CDC			
	OR	95 % CI	B Coefficient	p-Value
Gender				0.334
Male	2.72	0.36–20.08	1.002	
Age	1.011	0.99–1.03	0.011	0.261
BMI, kg/m^2^	1.010	0.94–1.085	0.010	0.794
Smoker				0.894
Yes	0.952	0.46–1.96	−0.049	
ASA score				0.080
ASA≥3	1.785	0.93–3.42	0.580	
Comorbidities				
Cardiovascular disease	0.926	0.50–1.73	−0.077	0.809
Diabetes mellitus	0.373	0.09–1.60	−0.985	0.185
COPD	0.951	0.21–4.28	−0.051	0.947
Prostatectomy in anamnesis				0.309
Yes	1.970	0.53–7.27	0.678	
Duration of surgery, min	1.008	0.99–1.02	0.008	0.069
Bilateral hernia				0.975
Yes	1.018	0.34–3.04	0.018	
Type of surgery			−1.400	**<0.001**
TAPP	0.247	0.118–0.514		
Level of training				0.138
Surgical trainee	1.632	0.85–3.12	0.490	

## Discussion

This retrospective study found no influence of the level of training of the surgeon on postoperative outcome after inguinal hernia repair. Surgery done by a surgical trainee, always supervised by a board-certified surgeon, was not a risk factor for higher intra- or postoperative complications for the classical open Lichtenstein repair procedure or the laparoscopic TAPP approach. The TAPP approach seems so be a protective factor for postoperative complications based on the multivariate analysis. However, other studies have shown that minimally invasive inguinal hernia repair procedures can be performed safely by residents [19], 20].

The postoperative complication rates after inguinal hernia repair are low compared to those of other general or visceral surgical procedures, which seems to make it an exemplary surgical procedure for educating surgical trainees. In recent literature, the rates vary from under 1–10 % (all degrees of complication summarized) and depend on the type of surgery [21], 22]. Weyhe et al. showed that major complications classified as CDC three or higher only occurred in around 20 % of cases [23]. We observed a complication rate of 9.8 %, and the rate of major complications was only 1.8 % of patients, which is in accordance with recent literature.

In the univariate analysis, TAPP was identified as a protective factor, which is in line with recent literature. Ashrafi et al. showed that patients undergoing TAPP had significantly lower rates of seroma, hematoma, and wound infections compared to patients receiving a Lichtenstein repair [21]. In contrast, Scheuermann et al. found no difference in postoperative complications between the techniques [24]. Our analysis was done with all inguinal hernia repairs pooled, and TAPP was performed significantly more often by board-certified surgeons, so our study does not answer the question of whether TAPP performed by residents would be associated with an increased risk of complications.

A wide range of risk factors have been described for perioperative complications in inguinal hernia repair. Known risk factors include patient age, BMI, diabetes, smoking, and duration of surgery [23], 25]. Previous prostatectomy is a risk factor for males developing inguinal hernia [26], 27]. Some studies also describe it as a risk factor for increased perioperative complications in minimally invasive hernia repair [28]. In a meta-analysis, Kasakewitsch et al. showed that patients with a previous prostatectomy had increased operation times and a higher rate of postoperative complications [29]. On the other hand, the meta-analysis by Aiolfi et al. showed no increased risk for intra- and postoperative complications for minimally invasive surgery after prostatectomy [30].

Nonetheless, surgical trainees needed 24 min more time on average to perform a unilateral TAPP, which was statistically significant. For bilateral TAPP, the duration of surgery was 16 min longer for the surgical trainee group, but the difference was not statistically significant. For Lichtenstein repair, no difference was observed between the groups. This finding is consistent with other studies [11], 14].

Prolonged operative time could be a risk factor for postoperative SSI. Especially in conjunction with mesh implants, an SSI could lead to infection of the implanted mesh. Kohno et al. showed a significantly increase of SSI per 1 h increase in operating time [31]. Christou et al. discussed the types of mesh implants and types of hernia as risk factors for SSI [32]. They analyzed over 21,000 patients from the French Hernia Club Registry, and the SSI rate was 0.24 % for open repair and 0.19 % for minimally invasive repair. They did not find any significantly risk factors in their univariate regression analysis [32]. Additionally, they found no connection between SSI and the type of implanted meshes.

Since men make up most patients undergoing inguinal hernia repair, protecting the spermatic cord is of particular importance. In our cohort, we found 2 cases (0.5 %) of injuries to the spermatic cord. Both cases were operated on by board-certified surgeons. Damous et al. found no impact of bilateral hernia repair using the Lichtenstein procedure or TAPP on male fertility [33]. Overall, the complication of injury to the spermatic cord or the resulting testicular atrophy appears to be so rarely described in the literature that it was not included as single factor in the analyses within the scope of reviews and meta-analyses [24], 34], 35].

The rate of hernia recurrence was reported as 1.2 % for TAPP by Andresen et al. and 4.7 % by Pokorny et al. [34], 35] For the Lichtenstein procedure, the reported recurrence rates are between 0 and 9 % [21], 35]. For both methods, the reported recurrence rates have wide distributions. We observed a recurrence rate of 2.1 % for both methods, which is within the range reported in the literature, and there was no difference between the resident and board-certified surgeon groups.

Overall, however, during the study period, board-certified surgeons performed more than twice as many inguinal hernia repairs as residents. Unfortunately, this reflects the reality of surgical training [36], 37]. Also, this reflects to some extent insurance status of patients, since privately insured patients are uniformly operated by board-certified surgeons. Currently, hospitals must also pay attention to cost-effectiveness and efficiency. In particular, the extension of operating time, which can result from the involvement of assistant doctors in the operation, could pose an economic problem for many surgeries involving surgical education [38], [39], [40].

To successfully integrate surgical training into daily clinical practice, several conditions must be met. Notably, structured supervision by experienced surgeons, the consistent incorporation of simulation-based training (such as hernia models), and institutional support that aligns educational needs with operational efficiency are essential [41], 42]. A recent systematic review confirmed that simulation-based training improves technical proficiency while maintaining patient safety and reducing errors in real clinical settings [43]. Additionally, real-time instructor feedback has been shown to optimize learning curves in simulation environments, further enhancing readiness for actual procedures [44]. Such modern educational approaches may not only benefit medical students but could also be valuable in the context of specialist-level surgical training.

### Limitations

This study has some limitations. The data were collected retrospectively from a single center. The overall complication rate was low, so the conclusions of the statistical analyses, especially the retrospective analysis, must be viewed with caution. In addition, it is no longer possible to retrospectively determine with certainty how many steps of the operation were actively performed by the residents. It is possible that only partial steps were performed by residents, while the attending board-certified surgeons performed large parts of the operation.

## Conclusions

While the involvement of surgical residents leads to a longer operation time for laparoscopic inguinal hernia repair, there is no evidence suggesting an increased risk of postoperative complications. Both resident-performed and specialist-performed surgeries demonstrated low complication rates, which aligns with established standards for hernia-repair outcomes. Our findings reflect the real-world clinical and educational setting of a tertiary care center in Germany, where surgical residents predominantly perform open inguinal hernia repairs, while board-certified surgeons more frequently conduct minimally invasive procedures. Importantly, both approaches were proven to be safe when performed by residents under appropriate supervision. These results highlight the feasibility of implementing structured surgical training within routine clinical practice while ensuring both patient safety and high-quality resident education. To sustain surgical training in daily practice, healthcare institutions should implement structured curricula alongside mentorship, integrate simulation-based preparation, and ensure sufficient hands-on experience. Additionally, trainees should engage in specialized continuing education offered by professional societies to complement their clinical training.

## References

[1] Primatesta P, Goldacre MJ (1996). Inguinal hernia repair: incidence of elective and emergency surgery, readmission and mortality. Int J Epidemiol.

[2] Leistenhernienchirurgie DA-K, Niebuhr H, Kockerling F, Fortelny R, Hoffmann H, Conze J (2023). Inguinal hernia operations-always outpatient?. Chirurgie (Heidelb).

[3] Stahlman S, Fan M (2020). Incidence of inguinal hernia and repair procedures and rate of subsequent pain diagnoses, active component service members, U.S. Armed Forces, 2010–2019. MSMR.

[4] Simons MP, Aufenacker T, Bay-Nielsen M, Bouillot JL, Campanelli G, Conze J (2009). European Hernia Society guidelines on the treatment of inguinal hernia in adult patients. Hernia.

[5] Amid PK, Shulman AG, Lichtenstein IL (1994). Lichtenstein herniotomy. Chirurg.

[6] Arregui ME, Davis CJ, Yucel O, Nagan RF (1992). Laparoscopic mesh repair of inguinal hernia using a preperitoneal approach: a preliminary report. Surg Laparosc Endosc.

[7] O’Reilly EA, Burke JP, O’Connell PR (2012). A meta-analysis of surgical morbidity and recurrence after laparoscopic and open repair of primary unilateral inguinal hernia. Ann Surg.

[8] Widder A, Reese L, Lock JF, Wiegering A, Germer CT, Kindl GK (2023). Postoperative analgesics score as a predictor of chronic postoperative inguinal pain after inguinal hernia repair: lessons learned from a retrospective analysis. World J Surg.

[9] Bittner R, Bain K, Bansal VK, Berrevoet F, Bingener-Casey J, Chen D (2019). Update of guidelines for laparoscopic treatment of ventral and incisional abdominal wall hernias (International Endohernia Society (IEHS)): part B. Surg Endosc.

[10] Bittner R, Bain K, Bansal VK, Berrevoet F, Bingener-Casey J, Chen D (2019). Update of guidelines for laparoscopic treatment of ventral and incisional abdominal wall hernias (International Endohernia Society (IEHS))-part A. Surg Endosc.

[11] Fernandez-Alberti J, Mata L, Orrego F, Medina P, Bogetti D, Porto EA (2023). Laparoscopic inguinal hernia repair: impact of surgical time in the learning curve. Surg Endosc.

[12] Kockerling F, Berger D, Jost JO (2014). What is a certified hernia center? The example of the German hernia society and German society of general and visceral surgery. Front Surg.

[13] Kockerling F, Bittner R, Kraft B, Hukauf M, Kuthe A, Schug-Pass C (2017). Does surgeon volume matter in the outcome of endoscopic inguinal hernia repair?. Surg Endosc.

[14] Stevens A, Meier J, Balentine C (2022). Resident involvement in inguinal hernia repair is safe but associated with increased operative time. J Surg Res.

[15] Rehman HU, Iqbal A, Ahmad I, Fayyaz H, Ali L, Iqbal J (2024). Exploring the impact of risk factors, preventive measures, and management strategies on surgical site infections in general surgery: a comprehensive assessment. J Ayub Med Coll Abbottabad.

[16] Scigliano NM, Carender CN, Glass NA, Deberg J, Bedard NA (2022). Operative time and risk of surgical site infection and periprosthetic joint infection: a systematic review and meta-analysis. Iowa Orthop J.

[17] Cuschieri S (2019). The STROBE guidelines. Saudi J Anaesth.

[18] Dindo D, Demartines N, Clavien PA (2004). Classification of surgical complications: a new proposal with evaluation in a cohort of 6336 patients and results of a survey. Ann Surg.

[19] Christophersen C, Fonnes S, Andresen K, Rosenberg J (2023). Risk of reoperation for recurrence after elective primary groin and ventral hernia repair by supervised residents. JAMA Surg.

[20] de Geus SWL, Geary AD, Arinze N, Ng SC, Carter CO, Sachs TE (2020). Resident involvement in minimally-invasive vs. open procedures. Am J Surg.

[21] Ashrafi A, Kabiri A, Shayesteh Zadeh B, Sadri P (2024). Comparison of postoperative short-term complications and recurrence after one year between laparoscopic transabdominal pre-peritoneal (TAPP) and lichtenstein tension free repair on the treatment of primary unilateral inguinal hernia. World J Plast Surg.

[22] Bladin O, Young N, Nordquist J, Roy J, Jarnbert-Pettersson H, Sandblom G (2023). Learning curve in open groin hernia surgery: nationwide register-based study. BJS Open.

[23] Weyhe D, Tabriz N, Sahlmann B, Uslar VN (2017). Risk factors for perioperative complications in inguinal hernia repair - a systematic review. Innov Surg Sci.

[24] Scheuermann U, Niebisch S, Lyros O, Jansen-Winkeln B, Gockel I (2017). Transabdominal preperitoneal (TAPP) versus lichtenstein operation for primary inguinal hernia repair – a systematic review and meta-analysis of randomized controlled trials. BMC Surg.

[25] Lundstrom KJ, Sandblom G, Smedberg S, Nordin P (2012). Risk factors for complications in groin hernia surgery: a national register study. Ann Surg.

[26] Umeda K, Takeda T, Hakozaki K, Yasumizu Y, Tanaka N, Matsumoto K (2022). A low subcutaneous fat mass is a risk factor for the development of inguinal hernia after radical prostatectomy. Langenbecks Arch Surg.

[27] Otaki T, Hasegawa M, Yuzuriha S, Hanada I, Nagao K, Umemoto T (2021). Clinical impact of psoas muscle volume on the development of inguinal hernia after robot-assisted radical prostatectomy. Surg Endosc.

[28] Trawa M, Albrecht HC, Kockerling F, Riediger H, Adolf D, Gretschel S (2022). Outcome of inguinal hernia repair after previous radical prostatectomy: a registry-based analysis with 12,465 patients. Hernia.

[29] Kasakewitch JPG, da Silveira CAB, Lima DL, Rasador ACD, Kasmirski J, Eguchi M (2024). Is previous prostatectomy a risk factor for postoperative complications following minimally invasive inguinal hernia repair? A systematic review and meta-analysis. Surg Endosc.

[30] Aiolfi A, Bona D, Cali M, Manara M, Bonitta G, Cavalli M (2024). Is previous radical prostatectomy a contraindication to minimally invasive inguinal hernia repair? A contemporary meta-analysis. Hernia.

[31] Kohno S, Hasegawa T, Aoki H, Ogawa M, Yoshida K, Yanaga K (2022). Analysis of risk factors for surgical site infection and postoperative recurrence following inguinal and femoral hernia surgery in adults. Asian J Surg.

[32] Christou N, Ris F, Naumann D, Robert-Yap J, Mathonnet M, Gillion JF (2022). Risk factors for surgical site infection after groin hernia repair: does the mesh or technique matter?. Hernia.

[33] Damous SHB, Damous LL, Borges VA, Fontella AK, Miranda JDS, Koike MK (2023). Bilateral inguinal hernia repair and male fertility: a randomized clinical trial comparing lichtenstein versus laparoscopic transabdominal preperitoneal (TAPP) technique. Surg Endosc.

[34] Andresen K, Rosenberg J (2024). Transabdominal pre-peritoneal (TAPP) versus totally extraperitoneal (TEP) laparoscopic techniques for inguinal hernia repair. Cochrane Database Syst Rev.

[35] Pokorny H, Klingler A, Schmid T, Fortelny R, Hollinsky C, Kawji R (2008). Recurrence and complications after laparoscopic versus open inguinal hernia repair: results of a prospective randomized multicenter trial. Hernia.

[36] Huber T, Richardsen I, Klinger C, Mille M, Roeth AA, AsTerOi DSG (2020). See (n)one, do (n)one, teach (n)one: reality of surgical resident training in Germany. World J Surg.

[37] Bohnen JD, Chang DC, George BC (2021). Operating room times for teaching and nonteaching cases are converging: less time for learning?. J Surg Educ.

[38] Hosler MR, Scott IU, Kunselman AR, Wolford KR, Oltra EZ, Murray WB (2012). Impact of resident participation in cataract surgery on operative time and cost. Ophthalmology.

[39] Mack J, Turner C, Carter D, Hallagan L, Whiting J, Falank C (2020). The effect of resident participation on appendectomy operative times. J Surg Educ.

[40] Schreckenbach T, Munch I, El Youzouri H, Bechstein WO, Habbe N (2019). The safety level of total central venous access port implantation performed by residents. J Surg Educ.

[41] Friedrich U, Backhaus J, Zipper CT, Konig S, Mavroveli S, Wiegering A (2020). Validation and educational impact study of the NANEP high-fidelity simulation model for open preperitoneal mesh repair of umbilical hernia. Hernia.

[42] Zipper CT, Friedrich U, Backhaus J, Konig S, Mavroveli S, Wiegering A (2020). Incisional hernia repair in a high-fidelity silicone model for open retro-muscular mesh implantation with preparation of the fatty triangle: validation and educational impact study. Hernia.

[43] Shahrezaei A, Sohani M, Taherkhani S, Zarghami SY (2024). The impact of surgical simulation and training technologies on general surgery education. BMC Med Educ.

[44] Laca JA, Kocielnik R, Nguyen JH, You J, Tsang R, Wong EY (2022). Using real-time feedback to improve surgical performance on a robotic tissue dissection task. Eur Urol Open Sci.

